# Online assessment of musical ability in 10 minutes: Development and validation of the Micro-PROMS

**DOI:** 10.3758/s13428-023-02130-4

**Published:** 2023-05-23

**Authors:** Hannah Strauss, Stephan Reiche, Maximilian Dick, Marcel Zentner

**Affiliations:** Department of Psychology, University of Innsbruck, Innsbruck, Austria

**Keywords:** Assessment, Music perception, Musicians, Musical ability, Musical aptitude, Psychometrics

## Abstract

We describe the development and validation of a test battery to assess musical ability that taps into a broad range of music perception skills and can be administered in 10 minutes or less. In Study 1, we derived four very brief versions from the Profile of Music Perception Skills (PROMS) and examined their properties in a sample of 280 participants. In Study 2 (*N* = 109), we administered the version retained from Study 1—termed Micro-PROMS—with the full-length PROMS, finding a short-to-long-form correlation of *r* = .72. In Study 3 (*N* = 198), we removed redundant trials and examined test–retest reliability as well as convergent, discriminant, and criterion validity. Results showed adequate internal consistency ($\overline{\omega}$ = .73) and test–retest reliability (ICC = .83). Findings supported convergent validity of the Micro-PROMS (*r* = .59 with the MET, *p *< .01) as well as discriminant validity with short-term and working memory (*r* ≲ .20). Criterion-related validity was evidenced by significant correlations of the Micro-PROMS with external indicators of musical proficiency ($\overline{r}$ = .37, *p*s < .01), and with Gold-MSI General Musical Sophistication (*r* = .51, *p*<.01). In virtue of its brevity, psychometric qualities, and suitability for online administration, the battery fills a gap in the tools available to objectively assess musical ability.

**Supplementary Information:**

The online version contains supplementary material available at 10.3758/s13428-023-02130-4.

## Introduction

Interest in musical ability has grown continuously over the past two decades (Zentner & Strauss, 2017). One reason for this development is the increasing corpus of studies indicating that musical ability is associated with a range of nonmusical abilities. This includes not only general auditory skills (Grassi et al., 2017), but also reading, phonological awareness, second language abilities, memory, executive functions, socio-emotional skills, and motor skills (see Thaut & Hodges, 2018; Sala & Gobet, 2020, for an overview of relevant studies). Beyond their relevance for basic research, a deeper understanding of these associations also holds the potential to inform treatment approaches to various conditions, such as dyslexia, dementia, or autism spectrum disorder (e.g., Boll-Avetisyan et al., 2020; Brancatisano et al., 2020; Lam et al., 2020; Marquez-Garcia et al., 2022).

The growth of studies on associations between musical capacities and mental or neural functioning has revived interest in the creation of tools for the objective measurement of musical abilities (Zentner & Gingras, 2019). This development is driven by several factors. First, batteries for the assessment of musical abilities can be helpful in particularizing the musical skills involved in nonmusical ability or impairment. For example, dyslexia appears to be primarily related to impairments in the perception and reproduction of rhythm, rather than to other musical deficits (Bégel et al., 2022; Boll-Avetisyan et al., 2020). Second, the ever-growing body of evidence on the benefits of music training often remains unclear as to whether outcomes are due to the music training itself or to preexisting individual differences in musical ability. Music ability tests can help disambiguate such findings. Third, even when outcomes can be attributed to musical intervention, it may remain unclear as to whether the outcome was driven by an improvement in musical skills or by nonmusical features of the intervention. In all these cases, objectively assessed musical skills can help rule out alternative explanations and enrich interpretation of the findings.

Although batteries for the assessment of musical abilities have been available for a century, in recent times, musical proficiency has more often been indirectly inferred from musicianship status rather than measured directly by standardized musical ability tests (Zentner & Gingras, 2019). One reason for this practice is that musical aptitude batteries created in the past century were primarily designed for use in educational contexts, such as for determining which children might be best suited for admission to music schools, learning an instrument, or playing in a band. Another reason relates to shortcomings of earlier batteries, such as outdated, unwieldy formats; deficiencies in stimulus design and control; and gaps in psychometric evaluation and documentation (see Zentner & Gingras, 2019).

In recognition of these limitations, over the past decade, investigators have developed a number of musical aptitude tests with better psychometric, acoustic, and practical properties (Zentner & Gingras, 2019). These advantages have led to increased use of musical ability batteries in domains that are concerned with music, notably psychology and neuroscience. Although this development is important in bolstering the credibility of relevant research findings, most current batteries assess a relatively narrow range of musical skills, usually skills in melody and rhythm discrimination (see Zentner & Gingras, 2019, for an overview). Because musical ability encompasses aspects beyond melody or rhythm perception, such batteries may have limited content validity. The Profile of Music Perception Skills (PROMS) was devised to assess a broader range of musical discrimination skills, including those in the domains of timbre, tempo, tuning, and nonmetric rhythm perception.

The PROMS exists in several versions that have all been shown to be both valid and reliable (Law & Zentner, 2012; Zentner & Gingras, 2019; Zentner & Strauss, 2017). Recent evidence includes demonstrations of large differences between musicians and nonmusicians on Mini-PROMS test scores (e.g., Sun et al., 2021; Vanden Bosch der Nederlanden et al., 2020), as well significant associations of PROMS-S scores with brain activation patterns involved in music processing (Rajan et al., 2021). Table 1 provides an overview of key characteristics of these versions, along with other recently developed musical ability tests. A more detailed overview of musical ability tests for general and special populations is provided in Table S1.

**Table 1:** Overview of recent musical aptitude tests assessing general musical perception ability

Name of test	Year	Subtest domain	Trials
MET	2010	M, R	104
SMDT	2014	M, R, P	63
PROMS	2012	A, ER, L, M, P, R, TB, TE, TU^a^	162
PROMS-S	2017	A, ER, M, P, R, TB, TE, TU	68
Mini-PROMS	2017	A, M, TE, TU	36

*Note.* Information compiled from Zentner and Gingras (2019).

*A* Accent, *ER* Embedded Rhythms, *L* Loudness, *M* Melody, *MET* Musical Ear Test, *Mini-PROMS* abridged version of the Profile of Music Perception Skills, *P* Pitch, *PROMS* Profile of Music Perception Skills, *PROMS-S* Profile of Music Perception Skills-Short, *R* Rhythm, *SMDT* Swedish Musical Discrimination Test, *TB* Timbre, *TE* Tempo, *TU* Tuning.

^a^A detailed description and illustration of the stimulus material used in the PROMS subtests is provided in Law and Zentner (2012).

A distinctive feature of the PROMS is that it has been optimized for online administration (Zentner & Strauss, 2017). This development has been motivated by the advantages offered by web-based data collection, including the ability to (a) reach more diverse samples, as well as rare or specific subpopulations; (b) recruit a larger number of participants, who provide higher statistical power; (c) conduct cross-cultural studies without significant recruiting challenges; and (d) run studies more quickly and inexpensively, especially when responses are scored and recorded automatically on the hosting platform. Finally, a test that can be administered online has great versatility as it can be administered in such diverse environments as a school, under individual testing conditions in a laboratory, or on adult participants’ personal computers outside the laboratory. These benefits became particularly obvious during the 2020–2022 pandemic, when in-person or laboratory testing was difficult or even impossible in many parts of the world.

Online implementation and delivery of the PROMS works through *LimeSurvey* (LimeSurvey GmbH n.d). *LimeSurvey* is a powerful, open-source survey web application that provides a great deal of flexibility for customizing online assessments by embedding JavaScript. This allows us, for example, to determine users’ operating systems and browsers in order to adapt the presentation of trials to user specifications, which minimizes risk of technical errors and ensures a stable test delivery environment. Researchers interested in using the PROMS for research purposes receive a research account that provides access to their own use of the PROMS.^1^

Although one of the strengths of the PROMS is that it allows assessment of music perception skills that are missing from other music aptitude batteries (e.g., discrimination skills in the domains of timbre, tuning, or tempo), sometimes investigators are primarily interested in obtaining an overall summative score of musical ability. This is likely to be the case, for instance, when musical aptitude is included as a control variable; when it is included as a secondary variable; when changes in performance over time need to be assessed for musical abilities overall rather than domain by domain; or when musical ability needs to be assessed for screening purposes. Furthermore, most current music aptitude batteries take at least 15 min to complete. When time with participants is limited, or when examining populations with limited attentional resources such as children or special populations, researchers may find that a battery that takes even 15 min is too long.

For these reasons, we sought to devise a musical test battery that could be administered in no more than 10 min, all while retaining the broad range of musical dimensions that is distinctive of the PROMS. The development of short versions of test batteries presents some challenges, however. First, although test trials or trials of a short version are usually all included in the full-length form, one cannot assume that the reliability and validity evidence of the full-length form automatically extends to the abbreviated form (Smith et al., 2000). As a consequence, it is essential to establish the reliability and validity of the new measure independently. Second, the examination of associations between the full-length and the abbreviated version requires the two versions to be administered separately to avoid inflated estimates resulting from correlated error variance.

Third, if the full-length version of a test has a multidimensional structure, the content validity of the short version’s overall score is contingent on preserving the diversity of the domains of the long version. Such preservation is undermined, for example, if item selection for the short version is one-sidedly based on statistical criteria, such as maximizing internal consistency. This can lead to overrepresentation of items from particular subscales that correlate more highly with the overall score than do items from other subscales, thereby reducing content validity (Kruyen et al., 2013; Smith et al., 2000). Thus, in shortening a test, researchers need to balance reliability and validity criteria, ideally attaining satisfactory reliability without sacrificing validity.

Fourth, because of these problems of construct under- or misrepresentation, extensive validation is particularly important in the case of abbreviated tests. This includes demonstrations of convergent validity, criterion validity, and discriminant validity. In the current case, this means that the instrument should show high correlation with other musical aptitude tests whose validity has already been established, and it should exhibit significant correlations with external indicators of musical proficiency. At the same time, the test should not be unduly related to generic, nonmusical skills of audition and cognition that a musical aptitude test might nonetheless inadvertently tax if not carefully designed.

## Overview of studies

In light of the preceding review and considerations, the overarching objective of the current research was to derive a battery from the full-length PROMS that could (a) be administered online in no more than 10 min, (b) retain the broadest possible range of musical dimensions characteristic of the original PROMS, and (c) provide a valid and reliable score of overall perceptual musical ability. To this end, we conducted three studies. In Study 1, we screened trials from the full-length PROMS for inclusion in four very brief versions of the PROMS and evaluated their properties regarding brevity, difficulty, reliability, and validity. In Study 2, the version providing the best trade-off between these properties—termed Micro-PROMS—was administered with the full-length PROMS to examine short-to-long version correlations and compare key psychometric properties of the two versions. In Study 3, we examined test–retest reliability, convergent validity of the battery with the Musical Ear Test, discriminant validity against short-term and working memory, and criterion validity with multiple separate indicators of musical proficiency.

## Study 1

### Method

#### Participants

Participants were 280 students (174 female, 106 male, 0 other) from the University of Innsbruck, aged 18 to 69 years (*M* = 24.35, *SD* = 7.70, *Mdn* = 22). Six (2.1%) participants considered themselves to be professional musicians, 43 (15.4%) semiprofessional musicians, 142 (50.7%) amateur musicians, 81 (15.4%) music-loving nonmusicians, and eight (2.9%) nonmusicians. Of those classified as amateur musician or above (*n* = 191), 150 reported that they were still practicing their instrument regularly, corresponding to a proportion of 54.6% of musically active participants against 45.4% of either nonactive amateurs or nonmusicians.

#### Creation of the Micro-PROMS

Trials for the Micro-PROMS were taken from all subtests of the full-length PROMS with the exception of the Loudness subtest, which had already been removed from previous PROMS versions because of its weak correlations with other subtests (Zentner & Strauss, 2017), and the Embedded Rhythm subtest, which would have required special instructions and practice trials, making the test longer than desired. The selection of trials was based on data from a sample of 667 participants, cumulated over seven different studies conducted both in the laboratory and remotely online. Trials were retained for inclusion according to general principles of item analysis, notably item difficulty, skewness, item-to-total correlation, and test–retest performance of individual trials. In this selection process, statistical criteria (e.g., high item-to-total correlations) were weighed against validity requirements (e.g., representation of the broadest possible range of musical dimensions of the PROMS) so as to achieve a good balance between reliability and validity.

Unlike previous PROMS versions, in which instructions and practice trials are specific to each of the subtests, we aimed at including only one general instruction and one set of practice trials to make the test more time-effective. The use of a single instruction also made it possible to present trials either in fixed sequence, wherein trials belonging to the same subtest were presented in successive order and the order of subtests was also fixed, or in random sequence, in which trials from any subtest could be preceded or followed by trials from any other subtest. In consideration of these aspects, we created four different trial sets, described in detail in the next section.

#### Measures

##### Micro-PROMS

The four versions of the new PROMS had the following characteristics: Version 1 included four trials from five subtests of the full-length PROMS (Melody, Tuning, Accent, Rhythm, and Timbre), resulting in 20 trials that were presented in fixed order. Version 2 was identical to Version 1, except that trials were presented in random order. Versions 3 and 4 included three to five trials from seven subtests of the full-length PROMS (the five subtests of Versions 1 and 2, plus trials from the Pitch and Tempo subtests), resulting in 23 trials that were presented in either fixed order (Version 3) or in random order (Version 4). For the fixed order versions, subtest order was balanced by alternating structural and sensory subtests (see Law & Zentner, 2012). For Version 1, trials grouped by subtest were presented in the following order: Melody, Timbre, Accent, Tuning, and Rhythm. For Version 2, the respective order was: Melody, Timbre, Tempo, Tuning, Rhythm, Pitch, and Accent. The characteristics of the four versions are summarized in the first three rows of Table 2.

As with all previous PROMS versions (see Law & Zentner, 2012; Zentner & Strauss, 2017), participants are presented a reference stimulus twice, separated by an interstimulus interval of 1.5 sec, followed by the comparison stimulus after an interval of 2.5 sec. The reference stimulus was presented twice to facilitate its encoding, thereby leaving less room for individual differences in memory capacity to affect the performance. Participants are asked to indicate whether reference and comparison are the same or different by selecting one of five answer options: “*Definitely same*,” “*Probably same*,” “*Probably different*,” “*Definitely different*,” and “*I don’t know.*” The distinction between “*probably*” and “*definitely*” was introduced in line with signal detection theory (Macmillan & Creelman, 2005; see also Hautus et al., 2021) to account for confidence level, whereas the “*I don’t know*” option was provided to reduce guessing. For each correct answer, participants received 1 point for high confidence ratings (i.e., “*Definitely same/different*”) and 0.5 points for lower confidence ratings (i.e., “*Probably same/different*”). Wrong answers and “*I don’t know*” answers were scored with 0 points.

As a commonly used paradigm in perceptual discrimination tasks, confidence ratings allow for finer sensory judgments compared to the traditional binary forced choice (Mamassian, 2020). Although there is evidence suggesting that answer format using confidence ratings rather than a yes/no answer format only affects results in terms of percent correct responses, and not in terms of $d^{\prime}$ or association with criteria (Goshorn & Goshorn, 2001), recently developed methods can fully account for confidence ratings (Aujla, 2022). One such measure is Vokey’s (2016) ${d}_p^{\prime }$, which is derived from fitting receiver operating characteristic (ROC) curves via principal component analysis and will be reported here as a more robust alternative to traditional sensitivity measures such as $d^{\prime}$ or *d*_*a*_*.*^2^ Some participants achieved hit and false-alarm rates of 0 or 1, indicating that they correctly identified either all or none of the stimuli of a given class (i.e., *same* or *different correct*) and confidence level. In line with the specialized literature, these values were adjusted using the 1/(*2N*) rule (Hautus, 1995; Macmillan & Kaplan, 1985).

We should note that throughout the analyses reported in this research, using raw scores, ${d}_p^{\prime }$ scores, or alternative *d*-prime measures such ${d}_{a}^{\prime}$ (Macmillan &  Kaplan, 1985) led to very similar findings and the same conclusions. Hence, we always report ${d}_p^{\prime }$ in the descriptive sections of the results but use raw scores for the correlational analyses for easier interpretation. The code for computing sensitivity estimates for the Micro-PROMS can be found under the OSF link provided at the end.

##### Music training

As in earlier studies that examined the PROMS (Law & Zentner, 2012; Zentner & Strauss, 2017), information on participants’ musical proficiency was assessed with multiple indicators: (a) self-rated level of musicianship (0 = nonmusician; 1 = music-loving nonmusician; 2 = amateur musician; 3 = semiprofessional musician; 4 = professional musician; (b) musical activity (0 = not playing an instrument or singing; 1 = used to play an instrument or sing but no longer practicing; 2 = regularly practicing an instrument or singing for 0–5 hours a week; 3 = regularly practicing an instrument or singing for 6–12 hours a week; 4 = regularly practicing an instrument or singing for 13–30 hours a week; 5 = regularly practicing an instrument or singing for more than 30 hours a week); and (c) participants’ years of music training in relation to their age. Because the three variables were internally consistent (*ω =* .83), they were *z*-transformed and the mean across the three variables used as a composite index reflecting individuals’ extent of music training and music-making, henceforth termed “music training” for the sake of brevity.

#### Procedure

The test was administered autonomously online, hosted on *LimeSurvey* (version 2.64.1+; LimeSurvey GmbH n.d). By embedding a JavaScript code, users’ operating systems and browsers are recognized and test delivery is adapted accordingly. This ensures stable test delivery regardless of the device or browser being used, and it also greatly reduces susceptibility to technical problems, such as delayed stimulus delivery. The tool also allows researchers to track test-taking times for each of the test components.

The instructions asked participants to take the test in a quiet environment and to use headphones. After answering questions about their sociodemographic background and music training, a script randomly assigned participants to one of the four Micro-PROMS versions. Each version was preceded by a general instruction page, a sound calibration page to set the volume, and a practice trial to familiarize participants with the tasks. On average, the full sessions took 14.5 min to complete (*SD* = 4.2, *Mdn* = 13.9), whereas the duration of the battery itself was slightly under 10 min on average (*Mdn* = 9.8).

In light of the exploratory and preliminary nature of the study, two criteria were used to determine sample size. For item analyses, we sought to achieve a case-to-trial ratio of 3:1. For preliminary evidence regarding validity, we determined the sample size based on previous correlations between PROMS scores and external indicators of music training, which were in the range of .35–.50 (Law & Zentner, 2012; Zentner & Strauss, 2017). Analyses using the R package *pwr* (version 1.3-0; Champely, 2020) indicated that a total of *N* = 244 (*n* = 61 per group) would suffice for the present purposes.

### Results and discussion

A summary of the main characteristics and outcomes of each of the four test versions is presented in Table 2. Regarding internal consistency, the version in which trials from five PROMS subtests were presented in fixed order performed best (Version 1), followed by Version 2, 3, and 4. In light of our goal to retain as many subtests of the original PROMS as possible to ensure content validity, we retained Version 3 for further development (henceforth termed Micro-PROMS). We accepted the slightly lower internal consistency in view of the fact that some lesser-performing trials could be replaced in subsequent studies to attain higher internal consistency.

**Table 2:** Descriptive summaries of test performance for the four initial Micro-PROMS versions

Features	Version			
	1	2	3	4
Order of trials	Fixed	Random	Fixed	Random
Number of trials	20	20	23	23
Subtests	M, TU, A, R, TB		M, TU, A, R, TB, P, TE	
Number of participants	74	72	60	74
Mean test duration in min (*SD*)	9.39 (2.11)	9.48 (1.77)	10.11 (1.44)	10.41 (1.31)
Mean total score (*SD*)	14.52 (2.73)	14.86 (2.32)	15.23 (2.62)	15.39 (2.53)
Mean ${d}_p^{\prime }$ (*SD*)	1.85 (0.75)	1.94 (0.78)	1.77 (0.77)	1.77 (0.68)
Mean item-to-total correlation	.37	.32	.33	.32
Omega (*ω*)	.73	.64	.63	.61
% of trials answered correctly^a^	79.5%	81.1%	74.9%	78.9%
Correlation with music training	.53*	.43*	.43*	.39*

*Note*. *PROMS* Profile of Music Perception Skills, *M* Melody, *TU* Tuning, *A* Accent, *R* Rhythm, *TB* Timbre, *P* Pitch, *TE* Tempo.

^*a*^ Correct answers combined regardless of confidence level.

* All *p*s < .01.

## Study 2

Study 2 had three main objectives. First, we aimed at improving the psychometric properties of the version retained from Study 1 by replacing trials that had performed least well. Second, we examined the extent to which this very brief version would be correlated with the full-length 162-trial version of the PROMS (Full-PROMS). Third, we sought to broaden the empirical basis for evaluating the new instrument’s psychometric properties by gathering additional information about its validity. To this end, we administered a questionnaire designed to capture musical competence and music appreciation, expecting the Micro-PROMS to exhibit higher correlations with the competence than with the appreciation part of the questionnaire. To achieve these aims, we administered both versions of the PROMS and the questionnaire to a new sample of listeners.

### Method

#### Participants

Participants were 109 psychology students of the University of Innsbruck (36 male, 73 female, 0 other) aged 18–52 years (*M* = 23.23, *SD* = 5.37, *Mdn* = 22), who completed both the Micro-PROMS and the Full-PROMS and received course credit for participation. None of the participants considered themselves to be professional musicians, eight (7.3%) considered themselves to be semiprofessional musicians, 34 (31.2%) amateur musicians, 56 (51.4%) music-loving nonmusicians, and 11 (10.1%) nonmusicians. Of those classified as amateur musicians or above (*n* = 42), 25 reported that they still practiced regularly, corresponding to a proportion of 22.9% musically active participants against 77.1% participants that were either musically nonactive or nonmusicians.

#### Measures

##### Micro-PROMS

To rectify some of the psychometric inadequacies of the retained version, we removed eight trials because of their low difficulty (% correct > 90%) and comparatively low item-to-total correlations. The trials were replaced by four trials from Version 1 examined in Study 1 and by one trial taken from the original Full-PROMS, all of which had performed well psychometrically. Also, subtest order was altered slightly from the one in Study 1 to achieve a better balance of subtests with differing number of trials. Subtests were presented in the following order: Melody, Tuning, Timbre, Tempo, Rhythm, Pitch, and Accent.

##### Full-PROMS

The PROMS includes nine subtests with 18 trials each for a total of 162 trials. In the original publication, internal consistency of the total score was *ω* = .95, test-retest was *ICC* = .88, and, on average participants had scored 109.60 points (*SD* = 17.88, see Law & Zentner, 2012).^3^

##### Music-Mindedness Questionnaire

The Music-Mindedness Questionnaire (MMQ, Zentner & Strauss, 2017) comprises a four-item Music Competence scale (e.g., “I can tell when an instrument is out of tune”) and a four-item Music Appreciation scale (e.g., “Musical experiences belong to the most precious experiences in my life”), each rated on a five-point scale (1 = “not at all”; 5 = “very much”)*.* In the current study, we used a slightly longer version of the MMQ, comprising seven items per scale. Internal consistency in the current study was *ω* = .90 for the Music Competence scale and *ω* = .87 for the Music Appreciation scale.

##### Music training

Questions and coding regarding the participants’ music training and background were the same as those described in Study 1. The internal consistency of the music training composite score was *ω* = .86.

#### Procedure

The procedure was similar to that of Study 1. Participants were first administered the Micro-PROMS, followed by the MMQ and the Full-PROMS. Informed consent was obtained from all participants before assessment. We expected the correlation between the short and the long version to be *r* ≳ .50 and validity correlations with external indicators of musical proficiency to be *r* ≳. 30 (see Table 2). Power was again estimated using the R package *pwr* (version 1.3-0; Champely, 2020). Results showed that to detect effects of this size with a power of .80 (*α* = .05), a sample of at least 84 participants would be required. Differences in correlation coefficients were examined using the R package *cocor* (Diedenhofen & Musch, 2015), using Dunn and Clark’s *z* (1969). Sensitivity values were computed using the same approach as in Study 1.

### Results

#### Descriptive statistics and psychometric properties

The correlation between the Full-PROMS total score and the Micro-PROMS was *r =* .72, *p <* .001 (with Micro-PROMS items removed, *r* = .66, *p* < .001). An examination of correlations between the Micro-PROMS score and individual PROMS subtest correlations is also of interest, for the new battery was conceived to represent the broadest possible range of musical dimensions that is distinctive of the full-length PROMS. This would not be the case, for instance, if the Micro-PROMS exhibited much stronger correlations with certain subtests than with others. Thus, if the Micro-PROMS were to correlate highly with Timbre and Pitch but exhibit low correlations with Accent and Tempo, that would indicate that the Micro-PROMS primarily measures abilities in timbre and pitch discrimination, and does not sufficiently account for discrimination abilities in the domains of rhythm and tempo. Ideally, then, we should find that all subtests exhibit moderately strong correlations with the Micro-PROMS total score. As shown by the individual PROMS subtest correlations with the Micro-PROMS total score, this was indeed the case: *r* = .48 (Embedded Rhythms), *r* = .51 (Pitch, Tempo), *r* = .54 (Tuning), *r* = .56 (Timbre), *r* = .58 (Accent), *r* = .62 (Rhythm), *r* = .68 (Melody), all *p*s < .001.

Table 3 provides additional information related to the Micro-PROMS (left column) and the Full-PROMS (middle column), notably raw scores, ${d}^{\prime}$ scores, and internal consistency values. Overall, the key metrics of the Micro-PROMS were remarkably similar to those of the longer version. Of particular note was the increase in internal reliability to *ω =* .75 compared to the version retained from Study 1 (*ω* = .63). We found neither any significant differences for gender, nor any significant correlations between the PROMS total scores and age.

**Table 3:** Side-by-side comparison of descriptive statistics and psychometric properties of Micro-PROMS and Full-PROMS

Key metrics	Micro-PROMS	Full-PROMS
Number of trials	20	162
Mean item-to-total correlation	*r =* .40	*r =* .32
Reliability	*ω =* .75	*ω =* .95
Mean test duration in min (*SD*)	10.92 (3.07)	60.55 (13.35)
Mean raw score (*SD*)	11.96 (3.20)	105.16 (19.09)
Mean % of trials answered correctly^a^	68.4%	73.8%
Mean skewness (*SD*)	−0.47 (1.00)	−0.97 (2.00)
Mean kurtosis (*SD*)	−0.46 (1.49)	3.51 (15.07)
Mean ${d}_p^{\prime }$ (*SD*)	1.23 (0.77)	1.57 (0.44)
MMQ Music Competence	*r* = .62**	*r* = .60**
MMQ Music Appreciation	*r* = .31**	*r* = .24*
Music training composite	*r* = .47**	*r* = .42**

*Note*. *N* = 109. *PROMS* Profile of Music Perception Skills, *MMQ* Music-Mindedness Questionnaire.

^*a*^ Correct answers combined regardless of confidence level.

*p < .05. **p < .01.

#### Preliminary evidence for validity

To obtain initial validity information, we examined associations of the Micro-PROMS total score with the Music Competence and Music Appreciation subscales of the MMQ. Because the PROMS measures musical ability rather than music appreciation, we expected test scores to be more strongly related to the MMQ-Competence scale than to the MMQ-Appreciation scale. As anticipated, the correlation of the Micro-PROMS with MMQ-Competence was significantly higher than with MMQ-Appreciation (*r* = .62 vs. *r* = .31, *z* = 3.71, *p* < .001).

To particularize the unique variance associated with each of the scales, we ran a multiple linear regression with the Micro-PROMS score as the outcome variable and the two MMQ-scales as predictors. When both variables were entered simultaneously, only the competence scale maintained a significant association with the Micro-PROMS (*β* = 0.60, 95% CI [0.43, 0.77], *p* < .001), whereas the appreciation scale ceased to explain variance in Micro-PROMS test scores (*β* = 0.04, 95% CI [−0.13, 0.21], *p* = .639). In further support of validity, the Micro-PROMS was strongly correlated with the music training composite score representing external indicators of musical proficiency (*r =* .47, *p <* .001).

### Discussion

Study 2 provided further evidence in support of the psychometric soundness of the Micro-PROMS. The high intercorrelation between the Micro-PROMS and the Full-PROMS indicates that the total score of the Micro-PROMS provides a reasonable approximation to the total score of the full-length PROMS. This finding is noteworthy in light of the drastic reduction of trials from 162 to 20 and the fact that the two versions of the battery were administered separately. Moreover, the changes to trials relative to the version retained from Study 1 led to satisfactory internal consistency. Validity correlations were similar to those that were obtained with the Full-PROMS. For example, the correlation between the music training composite and the Micro-PROMS was *r* = .47, which is not significantly different from the *r* =.42 correlation found for the Full-PROMS (*z* = 0.73, *p* = .463). Finally, content validity was evidenced by similar-sized and substantial correlations between the Micro-PROMS and all of the subtests of the Full-PROMS. This evidence is important in light of our goal to create an instrument that would be capable of reflecting the broadest possible range of musical skills that can be assessed with the original full-length PROMS.

Despite these encouraging results, not all trials performed equally well, suggesting that some lesser-performing trials could be removed to further shorten the battery. Moreover, the case for validity was limited to correlations with participants’ self-report of musical behavior and competence. A stronger case for validity could be made if the predicted pattern of convergent and discriminant validity correlation could be replicated on the basis of objective tests.

## Study 3

The goal of Study 3 was threefold—first, to replace and/or remove trials that had not worked well in Study 2 and to re-examine the psychometric properties of the battery; second, to evaluate the test–retest reliability of the battery; and third, to expand the basis for evaluating the validity of the battery. Convergent validity was examined against the Musical Ear Test (MET), an established battery of musical ability in melody and rhythm perception (Wallentin et al., 2010), whereas two composite indices of musical training and expertise, including the Goldsmiths Musical Sophistication Index (Gold-MSI), served as indicators of criterion validity.

Discriminant validity was examined against the Digit Span (DS) test, which measures both short-term and working memory (Groth-Marnat et al., 2001). Furthermore, we examined differential patterns, expecting stronger associations between the Micro-PROMS and scales tapping into music training or competence (e.g., MMQ-Competence; Gold-MSI perceptual abilities) than scales relating to music appreciation or engagement with music (e.g., MMQ-Appreciation; Gold-MSI emotions).

### Method

#### Participants

A total of 198 participants (77 male, 120 female, 1 other), aged 15–59 years (*M* = 23.89, *SD* = 6.45, *Mdn* = 22), fully completed the Micro-PROMS as well as the MET. Of those participants, 196 (99.0%) also completed the MMQ, 165 (83.3%) the DS test, and 105 (53.0%) the Gold-MSI.^4^ A total of 32 participants took part in a retest session conducted in the laboratory after completing the full test battery.

Overall, 14 participants (7.1%) considered themselves as nonmusicians, 79 (39.9%) each as music-loving nonmusicians and amateur musicians, 25 (12.6%) as semiprofessional musicians, and 1 (0.5%) as professional musician. The majority of participants reported having either played an instrument or sung at some earlier point in their lives (*n* = 47, 23.7%) or that they were still practicing regularly (*n* = 87, 43.9%), compared to a third of participants (*n* = 64) that reported having never been musically active in their lives.

#### Measures

##### Micro-PROMS

To improve the psychometric efficiency of the instrument, we removed three trials that had not added to reliability or validity. Furthermore, the Timbre subtest of the full-length PROMS had been represented by only one trial in the version retained for Study 2. To ensure that each of the seven subtests of the original PROMS was represented by at least two trials, we added a trial from the Full-PROMS Timbre subtest. As can be seen from Table 4, some musical dimensions were represented with two items, and some with three items. This was done intentionally to achieve a balance of “sequential” and “sensory” components of musical perceptual ability, each represented with nine items. The distinction is based on factor analyses of original version of the PROMS, which distinguished two PROMS factors, with Accent, Rhythm, Melody loading on the sequential factor, and Pitch, Tempo, Timbre, Tuning on the sensory factor (Law & Zentner, 2012). Subtest order was the same as in Study 2.

**Table 4:** Descriptive and psychometric information for each of the 18 Micro-PROMS items

Item number	Full-length PROMS^a^	Correct answer	Mean	*SD*	% Correct^b^	RIT	RIR	*ω* if item is dropped
1	M2	D	0.91	0.27	93	.41	.35	.70
2	M11	D	0.76	0.40	81	.46	.35	.70
3	M12	D	0.62	0.42	74	.31	.15	.69
4	TU12	D	0.24	0.38	32	.32	.18	.72
5	TU17	S	0.85	0.30	93	.30	.19	.73
6	TU18	D	0.57	0.45	66	.46	.31	.71
7	TB14	D	0.39	0.44	47	.48	.34	.71
8	TB16	S	0.84	0.30	93	.23	.12	.73
9	TE5	D	0.81	0.36	86	.29	.16	.73
10	TE12	D	0.60	0.43	71	.53	.40	.68
11	R4	S	0.55	0.45	65	.32	.16	.72
12	R12	D	0.88	0.30	92	.41	.31	.70
13	R18	D	0.78	0.38	83	.40	.27	.69
14	P10	D	0.37	0.44	45	.52	.40	.68
15	P12	D	0.33	0.42	41	.39	.24	.72
16	A3	D	0.81	0.35	87	.43	.33	.70
17	A5	S	0.68	0.42	77	.35	.22	.71
18	A12	D	0.49	0.46	57	.33	.16	.71

*Note.* RIT = item-total correlation (correlation between item score and overall test score); RIR = item-rest correlation (correlation between item score and overall test score without the given item); M = Melody; TU = Tuning; TB = Timbre; TE = Tempo; R = Rhythm; P = Pitch; A = Accent. D = Different; S = Same.

^a^ Item designation in the full-length PROMS. For example, the first item of the Micro-PROMS corresponds to Melody subtest item number 2 in the full-length PROMS, hence M2.

^b^ Percentage of correct answers regardless of confidence level.

##### Music training

Questions and coding regarding the participants’ music training were the same as described in Studies 1 and 2, comprising participants’ self-rated level of musicianship, musical activity, and years of music training adjusted for age. Internal consistency was *ω* = .71.

##### Music-Mindedness Questionnaire (MMQ)

As in Study 2, participants completed the MMQ, which assesses music competence and music appreciation. Both scales were internally consistent (*ω* = .85 and *ω* = .86, respectively).

##### Goldsmiths Musical Sophistication Index (Gold-MSI)

The Gold-MSI (Müllensiefen et al., 2014) is a multidimensional self-report questionnaire assessing participants’ musical sophistication on the basis of their active musical engagement, perceptual abilities, music training, singing abilities, and emotional responses to music. It comprises 38 items and is sensitive to individual differences in music sophistication in both musicians and nonmusicians (Müllensiefen et al., 2014). In the current study, we used the German translation by Schaal et al. (2014). Internal consistency for the subscales ranged from *ω* = .84 to *ω* = .89 and was *ω* = .91 for the overall score of musical sophistication.

##### Musical Ear Test (MET)

The MET (Wallentin et al., 2010) comprises a Melody and a Rhythm subtest with 52 trials each and takes approximately 20 min to complete (Correira et al., 2022). Participants are asked to judge whether two melodic or rhythmic phrases are identical or not. The MET has been shown to have high internal consistency (*α* = .87) and to be significantly correlated with other measures of musical expertise (Correira et al., 2022; Swaminathan et al., 2021; Wallentin et al., 2010). For the present study, an online version of the MET was implemented by the study authors using *LimeSurvey* (LimeSurvey GmbH n.d). Internal consistency was *ω* = .73 for the Melody subtest, *ω* = .75 for the Rhythm subtest, and *ω* = .85 for the total score.

##### DS test

The DS test is a widely used tool for measuring both short-term and working memory (see Groth-Marnat et al., 2001). Participants are presented with a list of numbers for a few seconds and are then asked to reproduce them from memory, either in the same order (forward DS) or in reverse order (backward DS). Lists successively increase in length and thus in difficulty. Assessment is stopped when participants fail to recall two consecutive lists of the same length. For the current study, the online version of the auditory DS test implemented in *Inquisit 4* (Millisecond Software, LLC, 2015) based on the procedure reported by Woods et al. (2011) was administered. We report two estimates each for forward and backward recall: (1) total trial (TT)—the TT-score reflects the total number of both correct and incorrect trials presented prior to two consecutive errors at the same list length; (2) maximum length (ML)—the ML-score reflects the maximum length of the list that was successfully recalled. While the TT-score is similar to the widely used total correct score obtained from the Digit Span subtest of the Wechsler intelligence tests, it shows poorer test–retest reliability and convergent validity than the ML-score (Woods et al., 2011).

#### Procedure

Similar to previous studies, instruments were administered online via *LimeSurvey* (version 2.64.1+; LimeSurvey GmbH n.d) and *Inquisit 4* (Millisecond Software, LLC, 2015). *Inquisit* was used to administer the DS test and required participants to install an app to enable remote assessment. Participants were recruited via a university mailing list, as well as through the subreddit *r/SampleSize* (see Shatz, 2017) and postings on the social media platforms Facebook and Instagram. Upon completion of the assessments, participants received individual feedback on their performance on the Micro-PROMS, the MET, and the DS test. Psychology students (*n* = 88, 44.4%) from the University of Innsbruck additionally received course credit for their participation. Participants were asked to complete the assessments in a quiet environment and to use headphones to minimize possible distractions.

Data collection took place in two stages (see Participants). After agreeing to an informed consent statement, participants were asked to provide information relating to sociodemographic and music background. After a sound calibration test, in stage 1 of the data collection, measures were completed in the following order: (1) Micro-PROMS, (2) MMQ, (3) MET, (4) DS test. In stage 2 of the data collection, the position of the Micro-PROMS, MET, and DS remained the same, and the Gold-MSI and MMQ were administered randomly in either second or fourth position of the sequence. After completion of the stage 2 assessments, participants were offered the opportunity to sign up for a retest session against an allowance of 10 EUR. Thirty-two individuals (16.2%) participated in the retest assessment, which took place in the laboratory. The time interval between the initial and the retest assessment was slightly over a week on average (*M* = 8.55, *SD* = 2.95, *Mdn* = 9).

From intercorrelations between the PROMS and other objective musical aptitude tests (Law & Zentner, 2012), associations between the MET and the Gold-MSI (Correira et al., 2022), and from findings of Studies 1 and 2, we expected convergent and criterion correlations of *r* >. 30. From the literature about associations between memory capacity and musical aptitude, we expected these associations to fall within the range of *r* = .20–.30 (e.g., Kunert et al., 2016; Swaminathan et al., 2021). As in the previous two studies, power was estimated using the R package *pwr* (version 1.3-0; Champely, 2020). Results showed that to detect correlations of this size with a power of .80 (*α* = .05), the current sample sizes were sufficient. Differences in correlation coefficients were examined using the R package *cocor* (Diedenhofen & Musch, 2015), using Dunn and Clark’s *z* (1969).

### Results

#### Descriptive statistics and reliability

On average, participants scored 11.08 (*SD* = 2.85; *Mdn* = 11) out of 18 points on the Micro-PROMS. The lowest score was 4.00 and the highest score was 17.50 points. For the MET, scores ranged between 51 and 98 points, with an average score of 77.84 (*SD* = 9.42, *Mdn* = 79) out of 104 points. Mean scores and variances were similar to those reported in a different online administration of the MET (Correira et al., 2022), indicating that the current test conditions and outcomes were comparable across the studies.

The average sensitivity of the Micro-PROMS, based on ${d}_p^{\prime }$ (see Study 1, Method), was *M* = 1.39 (*SD* = 0.78; *Mdn* = 1.33) and *M* = 1.32 (*SD* = 0.80; *Mdn* = 1.28) for initial and retest assessments, respectively (see Table 4 for detailed psychometric information for each of the 18 trials). Internal consistency was *ω* = .70 for the initial assessment and *ω* = .85 for the retest assessment. Test–retest reliability, computed using a two-way mixed-effects model (single ratings, absolute agreement), was ICC = 0.83, 95% CI [0.68, 0.92]. This value is very close to the test–retest figures obtained for the original version of the PROMS (Law & Zentner, 2012), as well as for the Short-PROMS and the Mini-PROMS (Zentner & Strauss, 2017).

#### Convergent, discriminant, and criterion validity

**Fig. 1:**
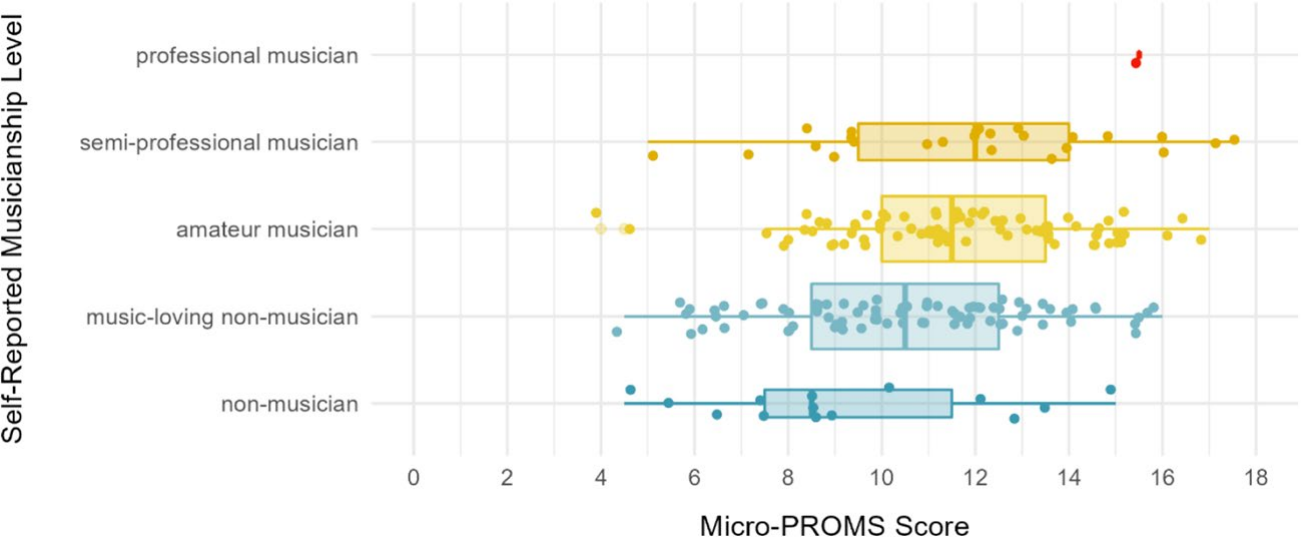
Distribution of Micro-PROMS total scores by different levels of self-reported musicianship status. *Note*. *N* = 198. Micro-PROMS scores differ as a function of self-reported musicianship level, *F*(4,193) = 4.47, *p* = .002

As shown in Table 5, the Micro-PROMS was highly and significantly correlated with the MET and with MMQ-Competence, lending support to its convergent validity. Discriminant validity was evidenced by significant but small correlations with DS forward, DS backward, and Gold-MSI Emotions. Furthermore, convergent correlations were significantly and markedly larger than discriminant correlations (see Table S2). Correlations were almost identical when controlled for participants’ sex and age. With regard to criterion validity, the Micro-PROMS total score was significantly associated with both our music training composite and the broader index of musicality provided by the Gold-MSI total score. Figure 1 depicts the scores of the Micro-PROMS for participants with different levels of self-reported musicianship, the latter representing one of the variables included in our music training composite (see Measures in Study 1).

**Table 5:** Means, standard deviations, and zero-order correlations of the Micro-PROMS and key validity measures

Variable	*M*	*SD*	*n*	1	2	3	4	5	6	7	8	9	10	11	12	13	14
1 Micro-PROMS	11.08	2.85	198														
2 MET Melody score	38.58	5.80	198	.57**													
3 MET Rhythm score	39.26	4.94	198	.45**	.54**												
4 MET total score	77.84	9.42	198	.59**	.90**	.85**											
5 DS forward (2-error max length)^a^	6.89	1.45	165	.16*	.30**	.15^+^	.26**										
6 DS backward (2-error max length)^a^	6.15	1.47	165	.23**	.25**	.25**	.29**	.42**									
7 MMQ Competence	2.99	0.86	196	.46**	.42**	.32**	.43**	.15^+^	.12								
8 MMQ Appreciation	3.39	0.88	196	.18*	.19**	.12^+^	.18*	−.10	−.15^+^	.48**							
9 Music training composite	−0.00	0.79	198	.26**	.39**	.16*	.33**	.11	.01	.75**	.42**						
10 Gold-MSI Active Engagement	3.68	1.10	105	.34**	.15	.35**	.28**	−.08	−.10	.49**	.69**	.37**					
11 Gold-MSI Perceptual Abilities	5.25	0.91	105	.45**	.29**	.38**	.38**	.06	.04	.76**	.38**	.59**	.38**				
12 Gold-MSI Singing Abilities	3.92	1.15	105	.46**	.41**	.25**	.38**	.09	.01	.66**	.40**	.54**	.27**	.60**			
13 Gold-MSI Musical Training	3.69	1.36	105	.40**	.49**	.39**	.51**	.08	−.05	.79**	.34**	.79**	.34**	.61**	.53**		
14 Gold-MSI Emotions	5.41	0.93	105	.22*	.12	.21*	.18^+^	−.31**	−.27**	.21*	.62**	.02	.51**	.30**	.22*	.09	
15 Gold-MSI General Musical Sophistication	3.99	0.98	105	.51**	.46**	.41**	.49**	.06	−.07	.86**	.56**	.77**	.61**	.75**	.79**	.82**	.33**

*Note*. DS = Digit Span; Gold-MSI = Goldsmiths Musical Sophistication Index; MET = Musical Ear Test; MMQ = Music-Mindedness Questionnaire; PROMS = Profile of Music Perception Skills.

^*+*^*p* < .10. *p < .05. **p < .01.

^a^ If DS scores were computed using the Total Trial instead of the Maximum Length scoring, correlations with the Micro-PROMS were *r*_*DS-forward*_ = .15* and *r*_*DS-backward*_ = .14 *ns*. Correlations with the MET total score were *r*_*DS-forrward*_ = .24** and *r*_*DS-backward*_ = .24**. MET-Subtest correlations were: MET-Melody with *r*_*DS-forward*_ = .26** and *r*_*DS-backward*_ = .21**; MET-Rhythm with *r*_*DS-forward*_ = .14 *ns.,* and with *r*_*DS-backward*_ = .21**.

Overall, the pattern of convergent and discriminant validity correlations of the Micro-PROMS and the MET were quite similar. For example, the Gold-MSI total score correlations with the Micro-PROMS and the MET did not differ significantly (*r* = .52 vs. *r* = .49, *z* = 0.35, *p* = .723). Moreover, the Micro-PROMS and the MET total score each explained independent amounts of variance in Gold-MSI General Musical Sophistication when entered simultaneously in a multiple regression (see Table 6). When the MET Melody and MET Rhythm subtests were entered separately, the Micro-PROMS was the sole significant predictor. As in previous studies, discriminant correlations of the Micro-PROMS with memory capacity were somewhat lower than those found for memory-to-MET correlations (Swaminathan et al., 2021; Wallentin et al., 2010).

**Table 6:** Summary of multiple regression analyses predicting music sophistication from MET and Micro-PROMS scores

	Gold-MSI General Musical Sophistication				
	*b*	*SE*	95% CI [LL, UL]	*β*	*p*
(Intercept)	0.31	0.65	[−0.97, 1.59]		.631
Micro-PROMS	0.12	0.04	[0.04, 0.20]	0.33	.003
MET (Total score)	0.03	0.01	[0.01, 0.05]	0.29	.007
Model Fit	*R*^*2*^ = .310, *R*^*2*^_*adj*_ = .296, *F*(2, 102) = 22.87 (*p* < .001)				
(Intercept)	0.33	0.65	[−0.96, 1.62]		.613
Micro-PROMS	0.12	0.04	[0.04, 0.20]	0.32	.003
MET (Melody score)	0.03	0.02	[−0.00, 0.07]	0.20	.069
MET (Rhythm score)	0.03	0.02	[−0.01, 0.06]	0.14	.187
Model Fit	*R*^*2*^ = .310, *R*^*2*^_*adj*_ = .290, *F*(3, 101) = 15.13 (*p* < .001)				

*Note*. *N* = 105. *Gold-MSI* Goldsmiths Musical Sophistication Index, *MET* Musical Ear Test, *PROMS* Profile of Music Perception Skills.

## Discussion

Taken together, these results offer solid evidence that the Micro-PROMS provides a reliable and valid assessment of musical ability, despite its short duration. Specifically, the instrument’s final 18-trial version proved internally consistent and also exhibited good test–retest reliability. Importantly, the Micro-PROMS met all validity criteria for successful test performance: convergent validity with the MET—a different, well-established battery of musical ability—as well as with a self-report instrument relating to musical competence; discriminant validity against short-term memory and working memory, and against self-report scales assessing emotional rather than ability components of musicality. The criterion validity correlations with the composite index of musical training was significant, if somewhat attenuated relative to the respective correlations reported in studies using the full-length PROMS and the Mini-PROMS (Law & Zentner, 2012; Zentner & Strauss, 2017), and those found in the present Studies 1 and 2. A possible explanation for the differences is provided by the near absence of professional musicians in the current sample (see Fig. 1) and the resulting range restriction in musicianship status. Still, the Micro-PROMS explained significant and substantial amounts of variance in Gold-MSI General Musical Sophistication, even when controlling for MET scores.

## General discussion

Across three studies involving over 580 participants, the current research introduced a test battery for the assessment of musical ability that has some distinctive features relative to earlier batteries. First, it is capable of providing an overall assessment of musical ability in about 10 min, making it the shortest test battery of overall perceptual musical ability that we are aware of. Second, it takes a broad range of music perception skills into account. In addition to tasks relating to discrimination for melody and rhythm, it includes trials relating to discrimination skills in the domains of pitch, timbre, tuning, tempo, and accent. Third, it has been devised for online administration that is easy for researchers and participants to use. In combining these features, the current measure goes an important step beyond previously existing measures toward meeting the requirement for a tool that can be used online to identify musical ability when time is critical.

### Psychometric properties

Against the aim of providing an overall summative score of musical ability in a very short time, the Micro-PROMS met the psychometric criteria of successful test performance. Specifically, despite using less than 15% of the trials of the full-length PROMS, the total scores of the two instruments were quite highly correlated and exhibited similar psychometric properties. Naturally, shortening a test battery to such an extent has costs. For example, the overall scores represent different levels of granularity, with the Micro-PROMS functioning as a screening tool for general musical aptitude, whereas the Full-PROMS provides a very detailed profile of multiple music perception abilities. Thus, the Micro-PROMS offers no domain-specific results, with the consequence that specific strengths and weaknesses cannot be assessed as is the case with the longer forms of the PROMS. All the same, the diversity of the contents represented by the subscales in the longer PROMS versions was preserved to some extent by including trials from nearly all subtests of the long version in the Micro-PROMS. Conceptually, then, the total score of the Micro-PROMS can be compared with the total score of the full-length PROMS.

Empirically, the comparability was evaluated against a number of psychometric measures. Internal consistency was adequate, if somewhat lower than that of the full-length PROMS. This was to be expected due to the Micro-PROMS’ small number of items capturing a wide range of musical content. In terms of validity, the pattern of correlations related to convergent, discriminant, and criterion validity was comparable to that of the full-length PROMS. More specifically, convergent and discriminant validity could be demonstrated against both objective ability tests (i.e., MET, DS) and self-report scales (Gold-MSI, MMQ). Criterion validity correlations with composite indices of musical training and expertise were significant and sizeable.

A comparison between the Micro-PROMS and the MET revealed that the two instruments perform about equally well in psychometric terms (see Correira et al., 2022; Swaminathan et al., 2021). In terms of reliability, the MET has slightly higher internal consistency than the Micro-PROMS, which was to be expected given the MET’s higher number of trials and stronger homogeneity in trial content. Because the test–retest reliability of the MET remains to be examined, test–retest reliability comparisons could not be drawn between the two instruments. With regard to validity, the size of correlations between the two batteries and the Gold-MSI were very similar, and similar also relative to MET-to-Gold-MSI correlations found in an earlier study with a large sample (Correira et al., 2022). In turn, the discriminant correlations of the Micro-PROMS with short-term and working memory have typically been lower, in the range of *r* ≈ .20 (e.g., Kunert et al., 2016; Vanden Bosch der Nederlanden et al., 2020), than those reported for the MET (Correira et al., 2022; Swaminathan et al., 2021; Wallentin et al., 2010; Zentner & Gingras, 2019). In the current study, the differences in correlations of the two instruments with memory outcomes were consistent with the earlier findings, if somewhat less pronounced.

The small associations between performance on the Micro-PROMS and memory tasks could be due to two distinctive aspects of the PROMS. First, trials assessing skills in domains such as timbre, pitch, and tuning are shorter and somewhat less complex than trials assessing rhythm and melody perception, taxing memory capacity less as a result. Thus, Talamini et al. (2016) found that only the Melody subtest of the Mini-PROMS was substantially correlated with auditory working memory. Second, in all versions of the PROMS, the reference stimulus is presented twice to facilitate its encoding, whereas in the MET and in other music aptitude batteries that we are aware of, the reference stimulus is presented only once. Both the shorter duration of the trials and the repetition of the reference stimulus seem to leave individual differences in memory skills little room to affect performance.

### Implications and uses

The Micro-PROMS will be particularly useful in situations where time is critical and researchers are primarily interested in a summative, overall estimate of musical ability. This may be the case, for example, when musical aptitude needs to be assessed as a secondary variable alongside several other constructs, when it is assessed as a control variable, or when the target sample is a special population with limited attentional resources (e.g., children, older adults, clinical samples). For the latter groups, the variations in trial content may be of additional help in sustaining attention and concentration. Brevity can be critical regardless of the target population, especially when investigators seek to obtain large and diverse samples. Thus, it has been found that the risk of dropout increases by up to 20% for each additional 10-min interval in web-based studies (Galesic & Bošnjak, 2009; see also Liu & Wronski, 2018).

Like previous versions of the PROMS, the Micro-PROMS was specifically devised for online administration. The process of making the PROMS suitable for online testing involved technical aspects, such as ensuring adaptability to variations in computer hardware, operating systems, and types of browsers, as well as ensuring that participants can take the test in the absence of an experimenter by formulating instructions that are clear and easy to follow. Furthermore, the settings allow researchers to provide automatically generated feedback of results displayed at the end of the test, which can be an incentive for participation.

Although the limited control over the participants’ listening environment is an understandable source of concern, research indicates that online and offline assessments of musical aptitude yield largely similar results (Correira et al., 2022). In our own research, we found that PROMS key metrics, such as internal reliability, trial difficulty, and validity correlations, obtained in the laboratory (Law & Zentner, 2012) and remotely online (Zentner & Strauss, 2017) were very similar. This finding is consistent with replications of data from in-person testing by data acquired online (e.g., Chetverikov & Upravitelev, 2016; Chierchia et al., 2019; Nussenbaum et al., 2020; Zentner & Strauss, 2017), including in the auditory domain (Milne et al., 2021).

Despite evidence suggesting that online assessments of musical ability are reliable, internet assessments will likely introduce a small amount of noise relative to in-person testing. Ultimately, the potential drawbacks of online testing need to be weighed against its advantages, such as the ease of reaching diverse samples, rare or specific subpopulations, or large numbers of participants who will in turn provide higher statistical power. Sometimes there is simply no choice, as has been the case in many parts of the world during the 2020–2022 pandemic. Researchers preferring to administer the Micro-PROMS via in-person or laboratory testing can easily do so, provided their work environments are connected to the internet.

### Limitations

Several limitations of the present investigation are noteworthy. First, although we found the Micro-PROMS to be psychometrically sound overall, additional studies are necessary to better establish its psychometric properties. For example, our samples were relatively homogeneous, and it is therefore necessary to examine the psychometric properties of the Micro-PROMS in samples of different ethnic and educational backgrounds, and across different age groups. The Micro-PROMS should be suitable for use in child and older adult populations because of its brevity, but the extent to which this is the case remains to be determined.

Second, it is important to keep in mind that the PROMS measures perceptual musical abilities. Although there is evidence to suggest that perceptual musical abilities are substantially correlated with certain musical production skills, such as tapping a tempo or rhythm (Dalla Bella et al., 2017; Georgi et al., 2023), current definitions of musicality encompass components such as abilities in the domains of performing or creating music (Levitin, 2012). The moderately strong correlations between performance on the PROMS and external indicators of musical proficiency, such as being a musician, are encouraging, but do not obviate the need for the construction of test batteries that tap into a broader array of musical talents.

Finally, although comparatively ample evidence for the battery’s convergent, discriminant, and criterion validity was obtained in the current studies, the validation of any test battery is a continuous process that will require different types of independent studies to produce definite results. The process might involve validation by examining associations with proximal indicators of musical behaviors, such as the ease with which musical novices acquire skills in understanding and/or producing music over time, or studies relating to distal criteria, i.e., nonmusical abilities that should nonetheless be conceptually related to musical aptitude, such as phonological awareness or vocal emotion recognition. Such information will be valuable but will take years to collect.

Despite its limitations, the Micro-PROMS closes an important gap in tools available for the assessment of musical ability. If a summative score of musical ability is all that researchers need, the Micro-PROMS represents an interesting alternative to longer versions of the PROMS or to other music aptitude batteries due to the broad array of music perception skills covered by the test, its brevity, and the ease with which it can be administered online.

### Supplementary Information

ESM 1 (DOCX 160 kb)

## Data Availability

The data and materials for all studies are available at https://osf.io/au6m5/. Administering the Micro-PROMS does not require a code, as it is freely accessible online. In order to request a PROMS research account please visit: https://musemap.org/resources/proms. None of the experiments was preregistered.

## Footnotes

1 The account offers researchers the possibility to tailor the online assessment to their needs, for example, by adding questionnaires that are of interest to the researcher. Researchers also have the option of providing participants feedback on their results at the end of the test. Responses can be accessed by researchers at any time and downloaded in multiple formats, including csv or SPSS files. The data are securely stored on university servers. The battery can be delivered in several languages. For more information, see: https://musemap.org/resources/proms/proms-user-guide

2 A small minority of participants (5.7%) only provided *definitely same* or *definitely different* answers. Due to the absence of less than fully confident answers, ${d}_p^{\prime }$ values could not be computed and were replaced with *d*-prime values computed from the individual hit and false-alarm rates of the collapsed rating categories using the same-different paradigm from the *psyphy*-package (v 0.2-3; Knoblauch, 2022; see also Hautus, 1995). Removing these participants did not change the results.

3 The “Loudness” subtest exhibited low correlations with all other PROMS subtests (Law & Zentner, 2012). It was therefore removed in the short version of full-PROMS, the PROMS-Short (see Zentner & Strauss, 2017). In the current study, it was administered for the sake of completeness, but not considered in subtest-level analyses.

4 A certain amount of attrition occurred for DS because test participants were asked to install special assessment software on their device. The Gold-MSI was added to the test battery at a second stage of data collection to expand the basis for examining content validity (see Procedure).

## Code availability (software application or custom code)

Code to compute *d*-prime estimates for the Micro-PROMS is available at https://osf.io/au6m5/.
