# Supplementary material for: Online assessment of musical ability in 10 minutes: Development and validation of the Micro-PROMS
Source: Behav Res Methods. 2023 May 23;56(3):1968–83. doi: 10.3758/s13428-023-02130-4 (PMC10991059; doi:10.3758/s13428-023-02130-4)
Supplement: Supplementary file 1 — (DOCX 160 kb) [file 13428_2023_2130_MOESM1_ESM.docx]

Supplemental Online Material for “Online Assessment of Musical Ability in 10 Minutes: Development and Validation of the Micro-PROMS”

**Table S1**

*Overview of Recent Musical Aptitude Tests Assessing General and Specialized Musical Perception Ability*

|  |  |  |  |  |  |  | **Psychometric information** | | | | | |
| --- | --- | --- | --- | --- | --- | --- | --- | --- | --- | --- | --- | --- |
| **Test** | **Format** | **Sample** | **Subtests** | **Trials** | **Duration** |  | **Reliability** | |  | **Validity** | | |
|  |  |  |  |  |  |  | **Internal consistency** | **Test-retest** |  | **Con-vergent** | **Discri-minant** | **Criterion** |
| **General Musical Perception Ability Tests (adapted from Zentner & Gingras, 2019)** | | | | | | | | | | | | |
| Gold-MSI (2014) | Offline / Online | adults | Melody, Beat-tracking | 30 | 15 min |  | x | x |  | - | - | x |
| MET (2010) | Offline^a^ | adults | Melody, Rhythm | 104 | 20 min |  | x | - |  | - | x | x |
|  | Online^b^ | adults | Melody, Rhythm | 104 | 20 min |  | x | - |  | - | - | x |
| PROMS (2012) | Online | adults | Melody, Rhythm, Embedded Rhythm, Tuning, Beat, Timbre, Tempo, Pitch, Loudness | 162 | 60 min |  | x | x |  | x | x | x |
| PROMS-S (2017) | Online | adults | Melody, Rhythm, Embedded Rhythm, Tuning, Beat, Timbre, Tempo, Pitch | 68 | 30 min |  | x | x |  | x | x | x |
| Mini-PROMS (2017) | Online | adults | Melody, Tuning, Beat, Tempo | 36 | 15 min |  | x | x |  | - | - | x |
| SMDT (2014) | Offline | adults | Melody, Rhythm, Pitch | 63 | 10 min |  | x | - |  | - | - | x |
|  |  |  |  |  |  |  |  |  |  |  |  |  |
| **Adaptive** **Musical Perception Ability Tests (LongGold test battery)** | | | | | | | | | | | | |
| CA-BAT (2018) | Offline / Online | adults | Beat | ~25 | 10 min |  | - | x |  | x | - | x |
| MPT (2018) | Offline / Online | Children (age 10+ years) & adults | Tuning | ~30 | 10 min |  | - | x |  | x | - | x |
| TPT (2020) | Offline / Online | adults | Timbre | 13 | 8 min |  | x | x |  | x | - | x |
| MDT (2017) | Offline / Online | adults | Melody | 10-20 / 20-35 | 3-6 min /  6-9 min |  | x | - |  | x | x | x |
|  |  |  |  |  |  |  |  |  |  |  |  |  |
| **Specialized Musical Perception Ability Tests** | | | | | | | | | | | | |
| BAASTA (2016) | Offline | healthy adults & patient populations | duration discrimination, anisochrony detection (with tones and music), Beat Alignment Test | ~ 71 + BAT | ~ 60 min (perceptual tasks) |  | - | x |  | - | - | x |
| META (2018) | Online | adult musicians | analytical hearing skills | 10-25 | 10-20 min |  | - | x |  | x | - | - |
| c-MDT (2018) | Offline | children (age 7-13 years) | Melody (perceptual task) | 40 | 20 min |  | x | - |  | - | x | x |
| MBEA (2003) | Offline | adults with amusia | Melodic Organisation, Rhythm, Meter, Melodic Memory | 184 | 60 min |  | - | x |  | x | - | x |
|  | Online^c^ | adults with amusia | Melodic Organisation, Rhythm, Meter, Melodic Memory | 72 / 184 | 15-30 min / 60 min |  | - | - |  | x | - | x |
| MBEMA (2013) | Offline | children (age 6-8 years) with amusia | Melodic Organisation, Rhythm, Meter, Melodic Memory | 60 | 20 min |  | - | - |  | x | - | x |
| CAMP (2009) | Offline | cochlear implant patients | pitch direction discrimination, melody recognition, timbre recognition | ? | 37 min |  | - | x |  | x | - | x |
|  |  |  |  |  |  |  |  |  |  |  |  |  |

*Note*. BAASTA = Battery for the Assessment of Auditory Sensorimotor and Timing Abilities (Dalla Bella et al., 2017); CA-BAT = Computerized Adaptive Beat Alignment Task (Harrison & Müllensiefen, 2018); CAMP = Clinical Assessment of Music Perception for Cochlear Implants (Kang et al., 2009); c-MDT = Children’s Melody Discrimination Task (Ireland et al., 2018); c-RST = Children's Rhythm Synchronization Task (Ireland et al., 2018); Gold-MSI = Goldsmith Musical Sophistication Index (Schaal et al., 2014); MBEA = Montreal Battery of Evaluation of Amusia (Peretz et al., 2003); MBEMA = Montreal Battery of Evaluation of Musical Abilities (Peretz et al., 2013); MDT = Melody Discrimination Test (Harrison et al., 2017); MET = Musical Ear Test (Wallentin et al., 2010); META = Musical Ear Training Assessment (Wolf & Kopiez, 2018); Mini-PROMS (Zentner & Strauss, 2017); MPT = Mistuing Perception Test (Larrouy-Maestri et al., 2019); PROMS = Profile of Music Perception Skills (Law & Zentner, 2012); PROMS-S = PROMS-Short (Zentner & Strauss, 2017); SMDT = Swedish Musical Discrimination Task (Ullén et al., 2014); TPT = Timbre Perception Test (Lee & Müllensiefen, 2020)

^a^ see also Swaminathan et al. (2021); ^b^ Correia et al. (2022); ^c^ see Pfeifer and Hamann (2015) for the full version and Peretz et al. (2008) for the short version

**Table S2**

*Dunn and Clark’s z (1969) and Respective p-Values for the Comparison of Correlations of Key Convergent and Discriminant Validity Measures with the Micro-PROMS Total Score*

|  |  | **MET** | **GOLD-MSI**  **General Sophistication** | **MMQ  Competence** |
| --- | --- | --- | --- | --- |
|  |  |  |  |  |
| **Discriminant Validity Measures** |  |  |  |  |
| DS forward (2-error max length) |  | 4.99** | 2.14* | 3.25** |
| DS backward (2-error max length) |  | 4.28** | 2.52* | 2.51* |
| DS forward (2-error total trials) |  | 4.88** | 2.03* | 3.35** |
| DS backward (2-error total trials) |  | 5.02** | 2.89** | 3.15** |
| MMQ Appreciation |  | 5.32** | 2.09* | 4.16** |
| Gold-MSI Emotion |  | 3.88** | 2.84** | 2.56* |
|  |  |  |  |  |

*Note*. Positive values indicate that the correlation coefficient *r* is larger for the measure displayed in the column than for the measure displayed in the respective row. For example, the first value relating to DS forward (2-error max length) indicates that the Micro-PROMS had a significantly larger correlation with the MET (convergent association) than with the forward digit span (discriminant association). DS = Digit Span (see Woods et al., 2011); Gold-MSI = Goldsmiths Musical Sophistication Index (Müllensiefen et al., 2014); MET = Musical Ear Test (Wallentin et al., 2010); MMQ = Music-Mindedness Questionnaire (see Zentner & Strauss, 2017); PROMS = Profile of Music Perception Skills.

**p*< .05. ***p*< .01.

**References**

Correia, A. I., Vincenzi, M., Vanzella, P., Pinheiro, A. P., Lima, C. F., & Schellenberg, E. G. (2022). Can musical ability be tested online? *Behavior Research Methods*, *54*(2), 955–969. https://doi.org/10.3758/s13428-021-01641-2

Dalla Bella, S., Farrugia, N., Benoit, C.‑E., Begel, V., Verga, L., Harding, E., & Kotz, S. A. (2017). Baasta: Battery for the Assessment of Auditory Sensorimotor and Timing Abilities. *Behavior Research Methods*, *49*(3), 1128–1145. https://doi.org/10.3758/s13428-016-0773-6

Harrison, P. M. C., Collins, T., & Müllensiefen, D. (2017). Applying modern psychometric techniques to melodic discrimination testing: Item response theory, computerised adaptive testing, and automatic item generation. *Scientific Reports*, *7*(1), 3618. https://doi.org/10.1038/s41598-017-03586-z

Harrison, P. M. C., & Müllensiefen, D. (2018). Development and Validation of the Computerised Adaptive Beat Alignment Test (CA-BAT). *Scientific Reports*, *8*(1), 12395. https://doi.org/10.1038/s41598-018-30318-8

Ireland, K., Parker, A., Foster, N., & Penhune, V. (2018). Rhythm and Melody Tasks for School-Aged Children With and Without Musical Training: Age-Equivalent Scores and Reliability. *Frontiers in Psychology*, *9*, Article 426, 426. https://doi.org/10.3389/fpsyg.2018.00426

Kang, R., Nimmons, G. L., Drennan, W., Longnion, J., Ruffin, C., Nie, K., Won, J. H., Worman, T., Yueh, B., & Rubinstein, J. (2009). Development and validation of the University of Washington Clinical Assessment of Music Perception test. *Ear and Hearing*, *30*(4), 411–418. https://doi.org/10.1097/AUD.0b013e3181a61bc0

Larrouy-Maestri, P., Harrison, P. M. C., & Müllensiefen, D. (2019). The mistuning perception test: A new measurement instrument. *Behavior Research Methods*, *51*(2), 663–675. https://doi.org/10.3758/s13428-019-01225-1

Law, L. N. C., & Zentner, M. (2012). Assessing Musical Abilities Objectively: Construction and Validation of the Profile of Music Perception Skills. *PLoS ONE*, *7*(12), e52508. https://doi.org/10.1371/journal.pone.0052508

Lee, H., & Müllensiefen, D. (2020). The Timbre Perception Test (TPT): A new interactive musical assessment tool to measure timbre perception ability. *Attention, Perception, & Psychophysics*, *82*(7), 3658–3675. https://doi.org/10.3758/s13414-020-02058-3

Müllensiefen, D., Gingras, B., Musil, J., & Stewart, L. (2014). The Musicality of Non-Musicians: An Index for Assessing Musical Sophistication in the General Population. *PLoS ONE*, *9*(2), e89642. https://doi.org/10.1371/journal.pone.0089642

Peretz, I., Champod, A. S., & Hyde, K. (2003). Varieties of musical disorders. The Montreal Battery of Evaluation of Amusia. *Annals of the New York Academy of Sciences*, *999*, 58–75. https://doi.org/10.1196/annals.1284.006

Peretz, I., Gosselin, N., Nan, Y., Caron-Caplette, E., Trehub, S. E., & Béland, R. (2013). A novel tool for evaluating children's musical abilities across age and culture. *Frontiers in Systems Neuroscience*, *7*, 30. https://doi.org/10.3389/fnsys.2013.00030

Peretz, I., Gosselin, N., Tillmann, B., Cuddy, L. L., Gagnon, B., Trimmer, C. G., Paquette, S., & Bouchard, B. (2008). On-line identification of congenital amusia. *Music Perception*, *25*(4), 331–343.

Pfeifer, J., & Hamann, S. (2015). Web-based testing of congenital amusia with the Montreal Battery of Evaluation of Amusia. In J. Ginsborg, A. Lamont, & S. Bramley (Eds.), *Proceedings of the Ninth Triennial Conference of the European Society for the Cognitive Sciences of Music (ESCOM)* (pp. 661–667). Royal Northern College of Music. https://www.fon.hum.uva.nl/silke/articles/pfeifer&hamann_2015_escom.pdf

Schaal, N. K., Bauer, A.‑K. R., & Müllensiefen, D. (2014). Der Gold-MSI: Replikation und Validierung eines Fragebogeninstrumentes zur Messung Musikalischer Erfahrenheit anhand einer deutschen Stichprobe. *Musicae Scientiae*, *18*(4), 423–447. https://doi.org/10.1177/1029864914541851

Swaminathan, S., Kragness, H. E., & Schellenberg, E. G. (2021). The Musical Ear Test: Norms and correlates from a large sample of Canadian undergraduates. *Behavior Research Methods*, *53*(5), 2007–2024. https://doi.org/10.3758/s13428-020-01528-8

Ullén, F., Mosing, M. A., Holm, L., Eriksson, H., & Madison, G. (2014). Psychometric properties and heritability of a new online test for musicality, the Swedish Musical Discrimination Test. *Personality and Individual Differences*, *63*, 87–93. https://doi.org/10.1016/j.paid.2014.01.057

Wallentin, M., Nielsen, A. H., Friis-Olivarius, M., Vuust, C., & Vuust, P. (2010). The Musical Ear Test, a new reliable test for measuring musical competence. *Learning and Individual Differences*, *20*(3), 188–196. https://doi.org/10.1016/j.lindif.2010.02.004

Wolf, A., & Kopiez, R. (2018). Development and Validation of the Musical Ear Training Assessment (META). *Journal of Research in Music Education*, *66*(1), 53–70. https://doi.org/10.1177/0022429418754845

Woods, D. L., Kishiyama, M. M., Yund, E. W., Herron, T. J., Edwards, B., Poliva, O., Hink, R. F., & Reed, B. (2011). Improving digit span assessment of short-term verbal memory. *Journal of Clinical and Experimental Neuropsychology*, *33*(1), 101–111. https://doi.org/10.1080/13803395.2010.493149

Zentner, M., & Strauss, H. (2017). Assessing musical ability quickly and objectively: Development and validation of the Short-PROMS and the Mini-PROMS. *Annals of the New York Academy of Sciences*, *1400*(1), 33–45. https://doi.org/10.1111/nyas.13410
